# Mechanism of Regulation of Big-Conductance Ca^2+^-Activated K^+^ Channels by mTOR Complex 2 in Podocytes

**DOI:** 10.3389/fphys.2019.00167

**Published:** 2019-02-28

**Authors:** Yinhang Wang, Jie Tao, Mengling Wang, Licai Yang, Fengling Ning, Hong Xin, Xudong Xu, Hui Cai, Weiguang Zhang, Ker Yu, Xuemei Zhang

**Affiliations:** ^1^Department of Pharmacology, School of Pharmacy, Fudan University, Shanghai, China; ^2^Department of Nephrology and Central Laboratory, Putuo Hospital, Shanghai University of Traditional Chinese Medicine, Shanghai, China; ^3^Department of Nephrology, Minhang Hospital, Fudan University, Shanghai, China; ^4^Renal Division, Department of Medicine, Emory University School of Medicine, Atlanta, GA, United States; ^5^Section of Nephrology, Atlanta Veteran Administration Medical Center, Decatur, GA, United States; ^6^Department of Nephrology, Chinese PLA General Hospital, Chinese PLA Institute of Nephrology, Beijing, China; ^7^Beijing Key Laboratory of Kidney Disease, State Key Laboratory of Kidney Diseases, National Clinical Research Center of Kidney Diseases, Beijing, China

**Keywords:** BK channels, mTORC2, Akt, SGK1, PKCα, podocyte

## Abstract

Podocytes, dynamic polarized cells wrapped around glomerular capillaries, are an essential component of the glomerular filtration barrier. BK channels consist of one of the slit diaphragm (SD) proteins in podocytes, interact with the actin cytoskeleton, and play vital roles in glomerular filtration. Mechanistic target of rapamycin (mTOR) complexes regulate expression of SD proteins, as well as cytoskeleton structure, in podocytes. However, whether mTOR complexes regulate podocyte BK channels is still unclear. Here, we investigated the mechanism of mTOR complex regulation of BK channels via real-time PCR, western blot, immunofluorescence, and patch clamping. Inhibiting mTORC1 with rapamycin or downregulating Raptor had no significant effect on BK channel mRNA and protein levels and bioactivity. However, the dual inhibitor of mTORC1 and mTORC2 AZD8055 and short hairpin RNA targeting Rictor downregulated BK channel mRNA and protein levels and bioactivity. In addition, MK2206, GF109203X, and GSK650394, which are inhibitors of Akt, PKCα, and SGK1, respectively, were employed to test the downstream signaling pathway of mTORC2. MK2206 and GF109203X had no effect on BK channel protein levels. MK2206 caused an obvious decrease in the current density of the BK channels. Moreover, GSK650394 downregulated the BK channel protein and mRNA levels. These results indicate mTORC2 not only regulates the distribution of BK channels through Akt, but also modulates BK channel protein expression via SGK1 in podocytes.

## Introduction

Big-conductance Ca^2+^-activated K^+^ (BK) channels, which couple intracellular electrical and chemical signals, are found in most excitable, as well as non-excitable cells, and play important roles in a variety of physiological functions, including regulation of neuronal firing, endocrine cell secretion, smooth muscle tone, and cellular proliferation and migration (Contreras et al., 2013; Pantazis and Olcese, 2016; Latorre et al., 2017; Maqoud et al., 2017; Dopico et al., 2018).

In the renal system, BK channels are highly expressed in nephrons and are important in K^+^ handling (Estilo et al., 2008). Recently, BK channels were discovered in human podocytes and their biological functions in mouse podocytes were described later (Morton et al., 2004; Grimm et al., 2007). To date, some podocyte proteins functionally related to the foot process cytoskeleton, such as nephrin, Nep1, the nephrin-like protein, and transient receptor potential cation channels, such as TRPC6, have been found to interact with BK channels directly and regulate the physiology and pathology of podocytes (Kim et al., 2008, 2009a,b, 2010). Abnormal expression of BK channels on the podocyte cell surface in some pathological conditions causes the destruction of cell actin cytoskeleton integrity, indicating that BK channels are essential for modulating the podocyte cytoskeleton (Kim and Dryer, 2011; Piwkowska et al., 2015). However, BK channels could promote Ca^2+^ influx through TRPC6, which maintains activation of podocyte BK channels (Kim et al., 2009a). Notably, BK channels are important in Ca^2+^ homeostasis in podocytes and influence glomerular filtration.

Recent research has shown that dysregulation of the mechanistic target of rapamycin (mTOR) has a role in certain common kidney diseases, such as diabetic nephropathy (Mori et al., 2009; Lu et al., 2011) and polycystic kidney disease (Perico et al., 2016). The mTOR protein complexes are serine/threonine kinases belonging to the phosphoinositide 3-kinase (PI3K)-related kinase family (Peng et al., 2017). Based on the different complex components Raptor and Rictor, the mTOR protein complexes are classified as mTORC1 and mTORC2, where mTORC1 contains mTOR, Raptor, mLST8, PRAS40, and Deptor and mTORC2 contains mTOR, Rictor, mSIN1, PRAS40, Protor-1, and Deptor (Gaubitz et al., 2016). These complexes are activated by nutrients, growth factors, cell stress, and phosphorylation (Yang et al., 2013). The mTORC1 is rapamycin sensitive and regulates cell growth, lipid biogenesis, and cell division by phosphorylating its substrates, including p70S6 Kinase (p70S6K), eukaryotic translation-initiation factor 4E-binding protein 1 (EIF4EBP1), Lipin1, and peroxisome proliferator-activated receptor gamma coactivator 1 alpha (PGC1-α) (Sancak et al., 2010). By contrast, mTORC2 is rapamycin insensitive and mainly modulates the downstream AGC kinase by phosphorylating its hydrophobic motif, such as through Akt, serum- and glucocorticoid-stimulated kinase (SGK), and protein kinases C (PKCs) (Jacinto et al., 2004; Ikenoue et al., 2008).

Few studies have reported on the functions of BK channels in podocytes and the underlying mechanisms remain elusive. Vollenbro et al. (2009) found inhibiting mTORC complexes reduces expression of slit diaphragm (SD) proteins, such as nephrin and TRPC6, and influences the cytoskeleton structure in podocytes by reducing the cytoskeleton adaptor Nck. However, the relationships between mTORC complexes and BK channels have not been studied. In addition, Eun Young Kim’s group investigated insulin modulation of BK channel activity associated with activation of Erk/MAPK and PI3K/Akt signaling pathways in podocytes (Kim and Dryer, 2011). Insulin, as a growth factor, can activate the PI3K/Akt signaling pathway, leading to activation of mTORC1 and mTORC2 (Saxton and Sabatini, 2017). LY294002, a PI3K inhibitor, can inhibit the insulin-induced increase in BK surface expression and macroscopic BK currents without altering the total BK protein expression levels (Kim and Dryer, 2011). However, whether mTOR complexes regulate BK channels in podocytes is unclear. Therefore, we speculated that the mTOR complexes may take part in regulating BK channels in podocytes. However, which mTOR complexes participate in regulating BK channels and how they mediate BK channels in podocytes has yet to be determined. In this study, we assessed the influence of mTORC1/2 on BK channels.

## Materials and Methods

### Cell Culture

Conditionally immortalized mouse podocytes, originally from Dr. Peter Mundel (Division of Nephrology, Massachusetts General Hospital, Harvard University), were kindly provided by Prof. Niansong Wang (Shanghai Sixth People’s Hospital, China). The podocytes were cultured as previously described (Mundel et al., 1997). Briefly, the podocytes were cultured at 33°C in RPMI1640 medium (Gibco, United States) with 10% fetal bovine serum (Gibco, United States), 100 U/ml penicillin-streptomycin (Gibco, United States), and 30 U/ml recombinant mouse interferon-γ (Sigma, United States). To induce podocyte differentiation, the cells were cultured at 37°C in medium lacking interferon-γ for 10–14 days. The methods of verifying phenotype after differentiation were provided in Supplementary Material.

### Real-Time RT-PCR

Total RNA was extracted using Trizol (TAKAR, AJapan) according to the manufacturer’s instructions. RNA concentration and purity were assessed using a TECAN infinite M200PRO Spectrophotometer. Total RNA (1 μg) was reverse-transcribed using PrimeScriptTM RT Master Mix (perfect Real Time; #RR036A, Takara, Japan) according to the manufacturer’s instructions. Each complementary DNA sample was analyzed in triplicate with a BIO-RAD CFX Connect Real-time PCR system using SYBR Premix Ex TaqTM (#RR0420A, Takara, Japan) with the following specific primers. Mouse KCa1.1 sense, 5′-GCGGCTTGAAGATGAGCAG-3′; mouse KCa1.1 antisense, 5′-TGCCAGGAATTAACAAGGGGT-3; mouse GAPDH sense, 5′-CGTCCCGTAGACAAAATGG-3′; and mouse GAPDH antisense, 5′-TCAATGAAGGGGTCGTTGA-3′.

### Western Blot

After different treatments, cells were lysed using NuPAGE-LDS sample buffer (Invitrogen, United States). Proteins were separated by SDS-PAGE and western blotting was carried according to standard procedures. Rabbit anti-KCa1.1 (1184–1200) antibody (APC-107) was purchased from Alomone Labs (Israel). Rabbit anti-Akt (ab32505), rabbit anti-p-Akt S473 (ab81283), rabbit anti-p-Akt T308 (ab38449), rabbit anti-β-actin (ab8227), rabbit anti-PKCα (ab32376), rabbit anti-p-PKC α S657/Y658 (ab23513), rabbit anti-SGK1 (ab43606), and rabbit anti-p-SGK1 S422 (ab55281) antibodies was purchased from abcam (United Kingdom). Rabbit anti-Raptor (#2280), rabbit anti-Rictor (#2114), rabbit anti-S6 (#2217), and rabbit anti-p-S6 Ser235/236 (#4868) antibodies were purchased from Cell Signaling Technology (United States). Band intensity was quantified using ImageJ software and normalized to the respective control.

### Lentiviral Production and Transfection

The pLKO.1 plasmids encoding mouse shRNA1 raptor (Addgene plasmid #21339) and shRNA1 rictor (Addgene plasmid #21341) were gifted by David Sabatini. The plasmids psPAX2 and PMD2G were kindly provided by Dr. Weijun WU (Department of Pharmacology, School of Pharmacy, Fudan University, China). Briefly, pLKO.1, shRNA1 raptor and shRNA 1 rictor plasmids were transfected into HEK293T cells together with plasmids psPAX2 and PMD2G to produce lentiviral particles by using Lipofectamine 3000 (Invitrogen) according to the manufacturer’s protocol. Cells were infected with lentivirus in presence of 5 μg/ml polybrene for 12 h. To achieve 100% positive cells, the cells were selected with 2 μg/ml puromycin after 2 days of virus transduction until the control cells died totally. Then, the transfected cells were lysed using NuPAGE-LDS sample buffer (Invitrogen, United States) for western blotting, analyzed for BK channel activity using patch clamping and analyzed for BK channel expression using immunofluorescence.

### Whole-Cell Patch Clamping

Whole-cell patch clamp recordings were conducted as described previously (Wu et al., 2016a,b) using an Axon Multiclamp 700B (Molecular Devices, United States) amplifier at room temperature. Patch pipettes were fabricated from glass capillary tubes using a PC-10 Puller (Narishige, Japan) with a resistance of 3–5 MΩ. Data acquisition and stimulation protocols were controlled by pCLAMP 10 (Molecular Devices, United States). Capacitance transients were canceled. Cells with a seal resistance (Rseal) of less than 1 GΩ were omitted. Series resistance (Rs) was compensated to 80–85% in order to minimize voltage errors. Cells with an uncompensated Rs higher than 10 MΩ were discarded. All patch-clamping experiments met this criterion.

Podocyte outward currents were elicited by step pulses ranging from -50 to +120 mV for 200 ms in increments of 10 mV. The holding potentials were held at -80 mV. The currents evoked using these protocols were entirely attributable to BK channels, which could also be elicited by exerting paxilline. To determine the voltage dependence of activation, the conductance was calculated using the formula: G(V) = I(V)/(V-ErK), where I(V) is the BK current at command voltage V and ErK is the reversal potential. The conductance was normalized to the maximal value and voltage dependence for activation of the BK channels fitted to the Boltzmann equation: f(x) = -1/{1+exp[(x-V1/2)/k]}+1, where V1/2 is the voltage at which half-maximal activation occurred and k describes the slope of the fit.

### Solutions and Drugs

The standard external solution for BK channels consisted of 150 mM NaCl, 5.4 mM KCl, 0.8 mM MgCl2, 5.4 mM CaCl2, and 10 mM HEPES and the pH of the solution was adjusted to 7.4 with NaOH. Internal solutions consisted of 10 mM NaCl, 125 mM KCl, 6.2 mM MgCl2, 10 mM HEPES, and 5 mM free Ca^2+^ and the pH of the solution was adjusted to 7.2 with KOH. The total Ca^2+^ was increased to yield the desired free concentration, which was calculated using the program Maxchelator^1^. Rapamycin (Selleck, United States), AZD8055 (Selleck, United States), MK2206 (Selleck, United States), GF109203X (Selleck, United States), and GSK650394 (Selleck, United States) were dissolved in DMSO (Sigma, United States) and the appropriate amount of DMSO was added to each control sample.

### Immunofluorescence

Podocytes were fixed in 4% paraformaldehyde in PBS for 15 min at room temperature. After rinsing twice with PBS, cells were blocked with 2% bovine serum albumen (BSA) in PBS for 1 h at room temperature. Cells on coverglass were incubated with KCa1.1 (1184–1200) antibody (APC-107, Alomone Labs) at a 1:100 dilution in PBS with 2% BSA at 4°C overnight. Cells were rinsed three times with PBS containing 0.02% Tween 20. Secondary antibodies were diluted in PBS with 2% BSA and cells were incubated at room temperature for 1 h. Alexa Fluor 488 goat anti-rabbit IgG (1:500; Cell Signaling Technology, United States) served as the secondary antibody. After rinsing three times with PBS containing 0.02% Tween 20, cells were incubated with DAPI (2 μg/ml) for 1 min. Cells were rinsed three times with PBS containing 0.02% Tween 20 and cells on coverglass were mounted on microscope slides with Prolong Gold antifade reagent (Invitrogen, United States). Images were taken using a Carl Zeiss LSM710 confocal microscope and processed using Photoshop software (Adobe Systems, Inc., San Jose, CA, United States). All of the images were quantified with ImageJ software and normalized to the respective control.

### Statistical Analysis

Electrophysiological data was analyzed using Origin 8.5 (OriginLab, United States) and expressed as mean ± SEM with the number of tests shown as *n* in the Results and Figure Legends. The other data were compared using independent samples *t*-tests, where data and images were analyzed using GraphPad Prism 5 software (San Diego, CA, United States). All A *p*-values less than or equal to 0.05 were considered statistically significant and all experiments were independently performed at least three times. Statistical comparisons were performed using unpaired two-tail *t*-tests (two group) or one-way ANOVA (>2 groups) where appropriate.

## Results

### Regulation of BK Channel Expression by mTORC1 in Podocytes

**FIGURE 1:**
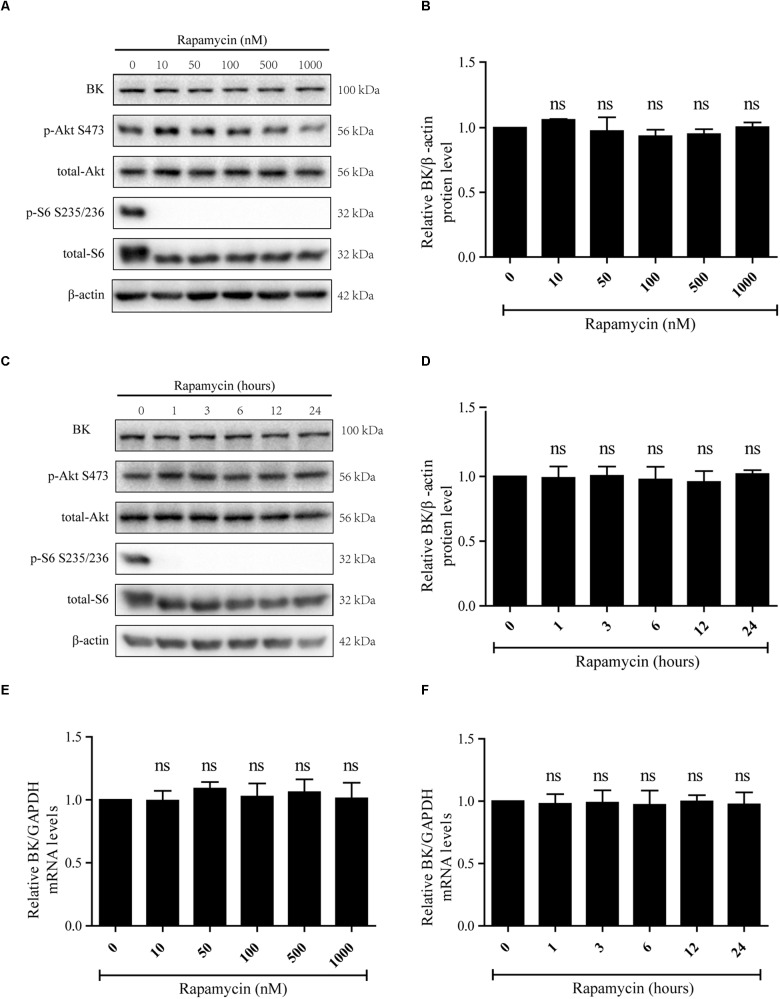
Effect of rapamycin on BK channel protein and mRNA levels in podocytes. **(A)** Podocytes were treated with 10, 50, 100, 500, or 1000 nM rapamycin for 24 h and then analyzed by immunoblot. **(B)** Quantification of BK channels protein expression by western blot. **(C)** Podocytes were treated with 50 nM rapamycin for 1, 3, 6, 12, or 24 h and then analyzed by immunoblot. **(D)** Quantification of BK channels protein expression by western blot. **(E)** Cells treated as in **(A)** were analyzed by real-time PCR. **(F)** Cells treated as in **(C)** were analyzed by real-time PCR. Data are expressed as mean ± SEM. ns, no statistical significance vs. control. One-way ANOVA and Dunnett’s Multiple Comparison test **(B–F)**.

Rapamycin, an mTORC1 inhibitor, was used to determine the effect of mTORC1 on BK channels. Podocytes were exposed to 10, 50, 100, 500, or 1000 nM rapamycin for 24 h or 50 nM rapamycin for 1, 3, 6, 12, or 24 h (Figure 1A–F). The concentrations and times were referred to Vollenbro et al. (2009) and Ding et al. (2014) studies. It was found p-Akt S473 and Akt levels were not significantly decreased compared with the control group (Figure 1A,C). Nevertheless, the p-S6 S235/236 levels were markedly decreased in all groups (Figure 1A,C). Rapamycin had no impact on BK channel mRNA levels (Figure 1E,F) and there was no difference in BK channel protein levels between the control and rapamycin treatment groups (Figure 1A–D).

### Inhibition of mTORC2 Decreases BK Channel mRNA and Protein Levels in Podocytes

**FIGURE 2:**
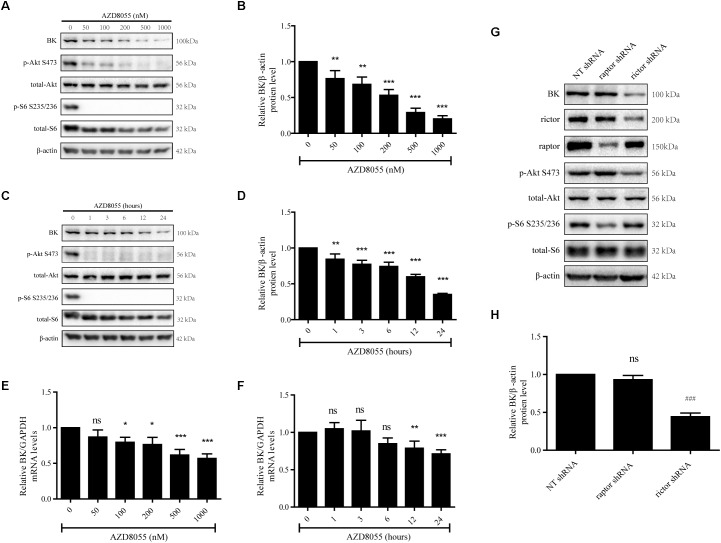
Effect of AZD8055 and Raptor- and Rictor-specific short hairpin RNA on BK channel expression in podocytes. **(A)** Podocytes were exposed to 50, 100, 200, 500, or 1000 nM AZD8055 for 24 h and then analyzed by immunoblot. **(B)** Quantification of BK channels protein expression by western blot. **(C)** Podocytes were exposed to 200 nM AZD8055 for 1, 3, 6, 12, or 24 h and then analyzed by immunoblot. **(D)** Quantification of BK channels protein expression by western blot. **(E,F)** Cells treated as in **(A,C)** were analyzed by real-time PCR. **(G)** Podocytes were transiently transfected with NT, Raptor, and Rictor short hairpin RNA (shRNA) and then analyzed by western blot. **(H)** Quantification of BK channels protein expression by western blot. Data are expressed as mean ± SEM. ^∗^*P* < 0.05 vs. control; ^∗∗^*P* < 0.01 vs. control; ^∗∗∗^*P* < 0.001 vs. control; ^###^*P* < 0.001 vs. NT shRNA; ns, no statistical significance. One-way ANOVA and Dunnett’s Multiple Comparison test **(B–H)**.

Because there is no mTORC2-specific inhibitor available, a dual inhibitor of mTORC1 and mTORC2, AZD8055, was used to evaluate the effects of mTORC2 on BK channel expression (Willems et al., 2012; Li et al., 2013). Podocytes were exposed to AZD8055 to assess the influence of mTORC2 on BK channels. We treated podocytes with 50, 100, 200, 500, or 1000 nM AZD8055 for 24 h (Figure 2A,E) or 200 nM AZD8055 for 1, 3, 6, 12, or 24 h (Figure 2C,F). The BK channel mRNA and protein expression levels displayed a significant decrease that was dependent on both concentration and time (Figure 2A–F).

To further verify these results, we used shRNAs specific for Raptor and Rictor to inhibit mTORC1 and mTORC2 activity, respectively. Meanwhile, the NT shRNA was set as negative control. Raptor and Rictor are subunits specific to mTORC1 and mTORC2 and, therefore, silencing of Raptor and Rictor has been used to block the activity of mTORC1 and mTORC2 in many studies. We found a significant decrease in BK channel protein expression when shRNA specific for Rictor, a component of mTORC2, was transfected into podocytes (Figure 2G). However, when Raptor shRNA was transfected, the BK channel protein expression levels did not change compared with the NT shRNA group (Figure 2G,H).

The data shown in Figure 1, 2 indicate inhibiting the activity of mTORC2, but not mTORC1, decreased BK channel mRNA and protein expression in podocytes.

**FIGURE 3:**
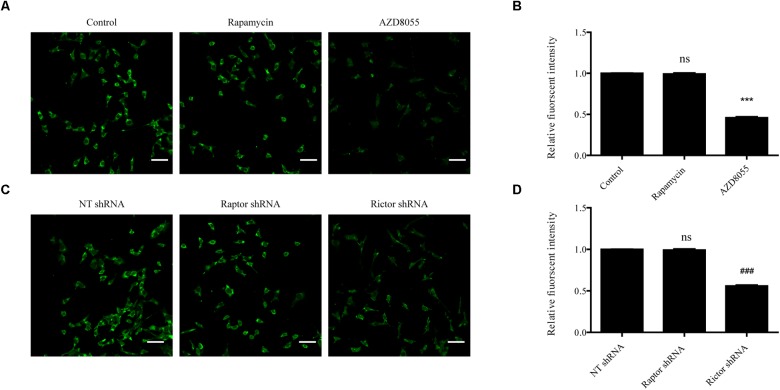
Influence of mTORC1 and mTORC2 on BK channel expression in podocytes. **(A)** Confocal microscopy of endogenous BK channels (green) in podocytes exposed to 50 nM rapamycin and 200 nM AZD8055 for 24 h. Scale bars, 50 μm, original magnification × 200. **(B)** Quantification of BK channels protein expression by immunofluorescence. **(C)** Confocal microscopy of endogenous BK channels (green) in podocytes transfected with NT, Raptor, and Rictor shRNA. Scale bars, 50 μm, original magnification × 200. **(D)** Quantification of BK channels protein expression by immunofluorescence. Data are expressed as mean ± SEM. ^∗∗∗^*P* < 0.001 vs. control; ^###^*P* < 0.001 vs. NT shRNA; ns, no statistical significance. One-way ANOVA and Dunnett’s Multiple Comparison test **(B,D)**.

To further determine the effect of mTORC2 and mTORC1 on the expression of BK channels in podocytes, podocytes were exposed to 200 nM AZD8055 and 50 nM rapamycin for 24 h. Confocal microscopy revealed a decrease in the fluorescent intensity of BK channels in podocytes in the AZD8055 group, but not the rapamycin group, compared with the control group (Figure 3A,B). Consistent with these results, there was a significant decrease in the fluorescent intensity of BK channels in podocytes transfected with shRNA specific for Rictor (Figure 3C,D). However, when Raptor shRNA was transfected into the cells, the fluorescent intensity of the BK channels remained unchanged compared with the NT shRNA group (Figure 3C,D).

### Inhibition of mTORC2, but Not mTORC1, Decreases the Bioactivity of BK Channels in Podocytes

**FIGURE 4:**
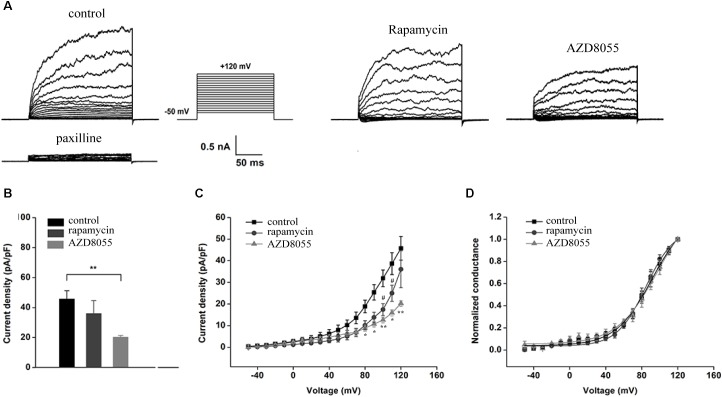
Effect of rapamycin and AZD8055 on the current density of endogenous BK channels in podocytes. **(A)** Representative traces of whole-cell currents from podocytes before and after application of 10 μM paxilline. The holding potential was -80 mV and the currents were evoked using step pulses ranging from -50 to +120 mV for 200 ms in increments of 10 mV and 5 μM free Ca^2+^ in the pipette solution. **(B)** Quantification of current densities. **(C)** Mean currents of control cells and cells treated for 24 h with 50 nM rapamycin and 200 nM AZD8055. **(D)** Plots of the normalized conductance as a function of command potential in the presence of 50 nM rapamycin and 200 nM AZD8055. Data are expressed as mean ± SEM. ^∗^*P* < 0.05 vs. control group; ^∗∗^*P* < 0.01 vs. control group; ^#^*P* < 0.05 vs. control group. One-way ANOVA and Dunnett’s Multiple Comparison test **(B,C)**.

The inhibitory effects of AZD8055 and rapamycin could also be observed in whole-cell recordings of endogenous BK channels in podocytes, which were obtained by administering recording electrodes filled with a pipette solution containing 5 μM free Ca^2+^ (Figure 4). Including micromolar concentrations of free Ca^2+^ in the recording pipettes allowed measurement of BK current density in response to depolarizing voltage steps from a holding potential of -80 mV. The outward currents of the podocytes could be almost completely blocked by paxilline at all membrane potentials (Figure 4A), which implies the main outward currents of podocytes are paxilline-sensitive BK channels. After a pre-exposure to 200 nM AZD8055 for 24 h, a decrease in BK channel current density was observed in podocytes at all membrane potentials from +80 to +120 mV (Figure 4A–C), but no significant change occurred upon application of 50 nM rapamycin on BK channel current density, except when the membrane potentials were set at +100 and +110 mV (Figure 4B, membrane potentials were set at +120 mV, control: 45.76 ± 5.44 vs. rapamycin: 36.06 ± 8.56, *P* > 0.05, *n* = 6; control vs. AZD8055: 20.26 ± 1.18, *P* < 0.01, *n* = 4). However, both rapamycin and AZD8055 had no effect on the voltage dependence or kinetics (Figure 4D) of BK activation in podocyte cell lines.

**FIGURE 5:**
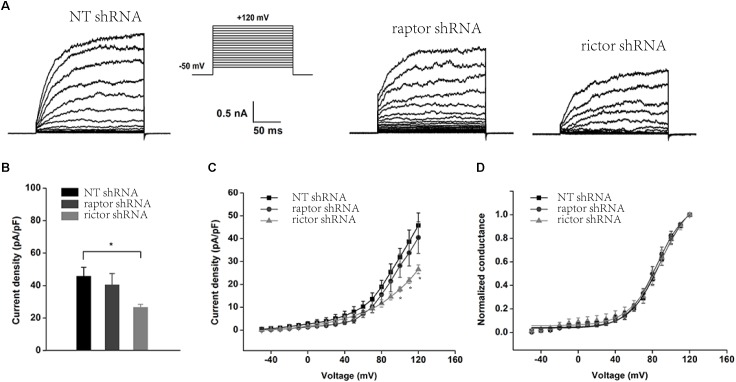
Effect of Raptor and Rictor shRNAs on the current density of endogenous BK channels in podocytes. **(A)** Representative traces of whole-cell currents from podocytes before and after application of 10 μM paxilline. The holding potential was -80 mV and the currents were evoked by step pulses ranging from -50 to +120 mV for 200 ms in increments of 10 mV and 5 μM free Ca^2+^ in the pipette solution. **(B)** Quantification of current densities. **(C)** Mean currents of cells transiently transfected with NT, Raptor, and Rictor shRNA. **(D)** Plots of the normalized conductance as a function of command potential in the absence of Raptor and Rictor. Data are expressed as mean ± SEM. ^∗^*P* < 0.05 vs. NT shRNA. One-way ANOVA and Dunnett’s Multiple Comparison test **(B,C)**.

Inhibition of mTORC2 and mTORC1 by knocking down Rictor and Raptor was also observed in whole-cell recordings of podocyte BK channels (Figure 5). The BK channel current density was obviously reduced in podocytes with stably transfected Rictor shRNA at membrane potentials from +100 to +120 mV (Figure 5A–C), but downregulation of Raptor had no effect (Figure 5B, the membrane potentials at +120 mV, NT shRNA: 48.17 ± 5.89; Raptor shRNA: 40.44 ± 6.92, *P* > 0.05, *n* = 6; Rictor shRNA: 26.55 ± 1.92, *P* < 0.01, *n* = 5). However, knocking down Rictor in conjunction with Raptor had no significant effects on the voltage-dependent kinetics (Figure 5D) of podocyte BK channel activation.

### Inhibition of p-Akt Decreases the Bioactivity of BK Channels in Podocytes

**FIGURE 6:**
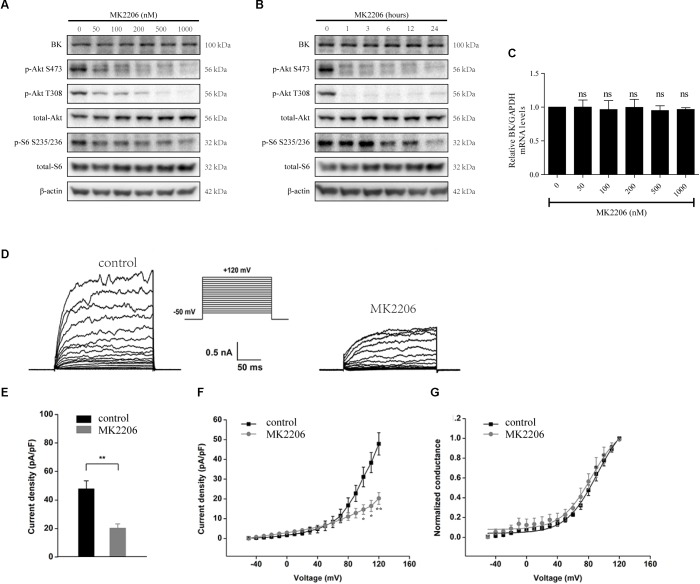
Effects of MK2206 on BK channels in podocytes. **(A)** Podocytes were exposed to 50, 100, 200, 500, or 1000 nM MK2206 for 24 h and then analyzed by immunoblot. **(B)** Podocytes were exposed to 200 nM MK2206 for 1, 3, 6, 12, or 24 h and then analyzed by immunoblot. **(C)** Cells treated as in **(A)** were analyzed by real-time PCR. **(D)** Representative traces of whole-cell currents from podocytes before and after application of 10 μM paxilline. The holding potential was -80 mV and the currents were evoked by step pulses ranging from -50 to +120 mV for 200 ms in 10 mV and 5 μM free Ca^2+^ in the pipette solution. **(E)** Quantification of current densities. **(F)** Mean currents of control cells and cells treated for 24 h with MK2206. **(G)** Plots of the normalized conductance as a function of command potential in the presence of MK2206. Data are expressed as mean ± SEM. ^∗^*P* < 0.05 vs. control; ^∗∗^*P* < 0.01 vs. control; ns, no statistical significance. One-way ANOVA and Dunnett’s Multiple Comparison test **(C)**, student’s *t*-test **(E,F)**.

MK2206, an Akt inhibitor, was used to evaluate the influence of Akt on BK channels (Bai et al., 2014; Hu et al., 2018). Podocytes were exposed to 50, 100, 200, 500, or 1000 nM MK2206 for 24 h or 200 nM MK2206 for 1, 3, 6, 12, or 24 h. We found MK2206 decreased the expression of p-Akt S473 and p-Akt T308 (Figure 6A,B). However, MK2206 had no influence on BK channel protein and mRNA levels (Figure 6A–C). These data indicate mTORC2 does not regulate BK channel protein expression through the Akt signaling pathway.

The effects of MK2206 were also observed in whole-cell recordings of podocyte BK channels (Figure 6D–G). After a pre-exposure to MK2206 for 24 h, a decrease in BK channel current density was observed in podocytes at membrane potentials from +100 to +120 mV (Figure 6E, membrane potential at +120 mV, control: 47.87 ± 5.66 vs. MK2206: 20.25 ± 3.00, *P* < 0.01, *n* = 4). However, MK2206 had no significant effect on the voltage dependence or kinetics (Figure 6G) of podocyte BK channel activation.

### Modulation of BK Channel Protein Levels by SGK1 in Podocytes

PKCα and SGK1 are two mTORC2 downstream substrates (Su and Jacinto, 2011). Therefore, we tested whether PKCα and SGK1 take part in regulation of BK channel expression in podocytes (Shibata et al., 2007; Lei et al., 2017). Because PKCα and SGK1 are directly phosphorylated at S657/Y658 and S422 by mTORC2, the levels of PKCα S657/Y658 and SGK1 S422 are regulated by mTORC2.

**FIGURE 7:**
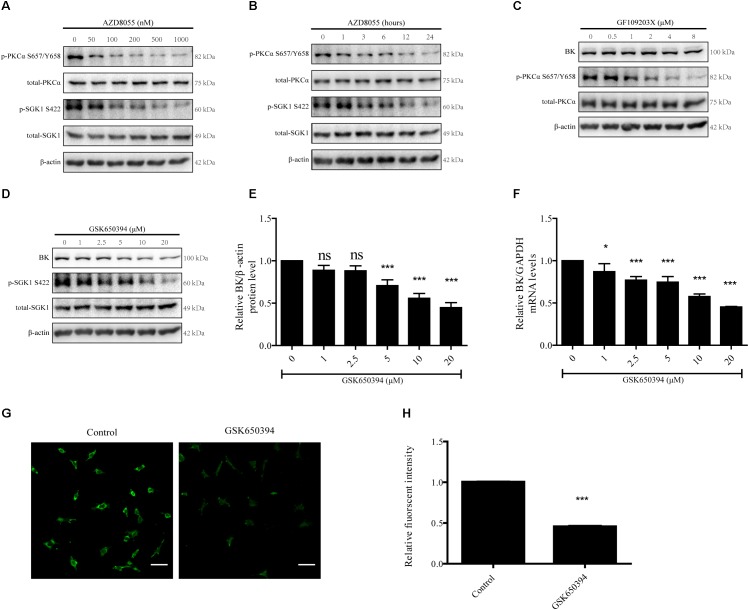
Effects of PKC and SGK-1 inhibitors on expression of BK channels in podocytes. **(A)** Podocytes were exposed to 50, 100, 200, 500, or 1000 nM AZD8055 for 24 h and then analyzed by immunoblot. **(B)** Podocytes were exposed to 200 nM AZD8055 for 1, 3, 6, 12, or 24 h and then analyzed by immunoblot. **(C,D)** Podocytes were exposed to 0.5, 1, 2, 4, or 8 μM GF109203X or 1, 2.5, 5, 10, or 20 μM GSK650394 for 24 h and then analyzed by immunoblot. **(E)** Quantification of BK channels protein expression by western blot of **(D)**. **(F)** Cells treated as in **(D)** were analyzed by real-time PCR. **(G)** Confocal microscopy showing endogenous BK channels (green) in podocytes treated with 10 μM GSK650394 for 24 h. Scale bars, 50 μm, original magnification × 200. **(H)** Quantification of BK channels protein expression by immunofluorescence. Data are expressed as mean ± SEM. ^∗^*P* < 0.05 vs. control; ^∗∗∗^*P* < 0.001 vs. control; ns, no statistical significance. One-way ANOVA and Dunnett’s Multiple Comparison test **(E,F)**, student’s *t*-test **(H)**.

To verify the effects of mTORC2 on PKCα and SGK1 in podocytes, podocytes were treated with various concentrations of AZD8055 for 24 h or 200 nM AZD8055 for different durations (Figure 7A,B). The p-PKCα S657/Y658 and p-SGK1 S422 levels displayed significant decreases that were concentration and time dependent (Figure 7A,B).

Next, we used GF109203X (bisindolylmaleimide I), a PKC inhibitor, to test whether PKCα takes part in regulating BK channel protein expression in podocytes (Lee et al., 2006; Ambrus et al., 2015; Yao et al., 2015). We treated podocytes with 0.5, 1, 2, 4, or 8 μM GF109203X for 24 h. We found GF109203X decreased the expression of p-PKCα S657/Y658, but had no influence on the BK channel protein levels (Figure 7C).

Finally, we used GSK650394, an SGK1 inhibitor, to inhibit SGK1 activity (Kook and Jones, 2016; Ding et al., 2017). As shown in Figure 7D–F, podocytes were exposed to 1, 2.5, 5 10, or 20 μM GSK650394 for 24 h to decrease p-SGK1 S422 levels and a significant decrease in BK channel protein and mRNA expression levels in a concentration-dependent manner was observed. To further determine the effect of SGK1 on BK channel expression in podocytes, podocytes were exposed to 10 μM GSK650394 for 24 h. Confocal microscopy revealed the fluorescent intensity of BK channels (green) in podocytes in the GSK650394 group was significantly decreased (Figure 7G,H). These data indicate mTORC2 regulates BK channel expression through SGK1.

## Discussion

**FIGURE 8:**
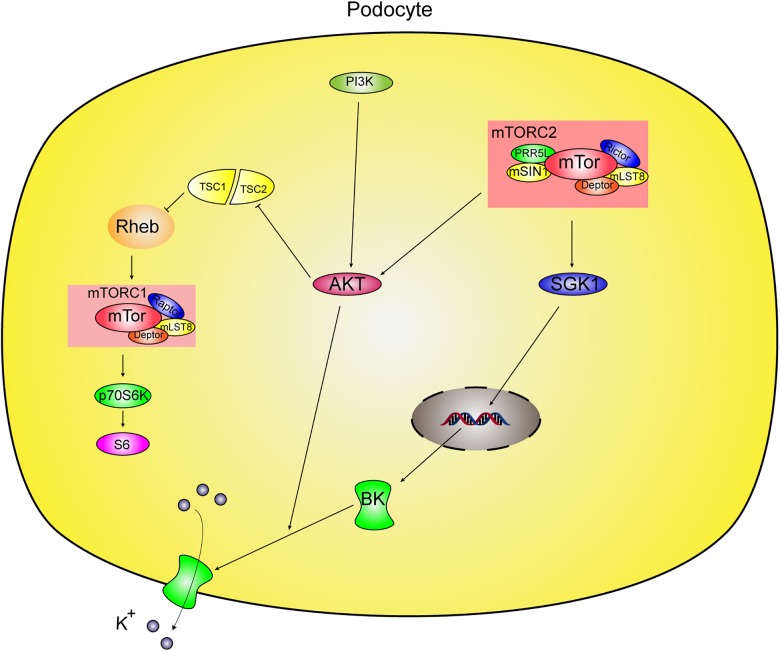
Proposed mechanism of regulation of BK channels by mTORC2 in podocyte. mTOR protein complexes contains mTORC1 and mTORC2 in podocytes. mTORC2 phosphorylates and activates its substrates, Akt and SGK1. mTORC2 modulates BK channels in podocytes via two different pathways. One way is mTORC2 mediates BK channel activity by influencing the surface expression of BK channels through Akt. The other is mTORC2 regulation of BK protein expression through SGK1 in podocytes. However, mTORC1 did not regulate BK in podocytes.

In this study, we investigated mTOR complex modulation of BK channels in podocytes and found novel modulation by mTORC2 of BK channels in podocytes via two different pathways (Figure 8). One pathway is through mTORC2 regulation of the distribution of BK channels through Akt and the other is through mTORC2 regulation of BK protein expression through SGK1 in podocytes.

The most important question addressed in this study is whether mTOR complexes take part in regulation of BK channels in podocytes. First, we used an mTOR inhibitor and shRNA to test regulation of BK channels by mTORC complexes in podocytes. P-S6 phosphorylated by p70S6K is widely used to indicate mTORC1 activation in a lot of studies. Because of directly phosphorylation at Ser473 by mTORC2, p-Akt Ser473 is often used to demonstate the activation of mTORC2. In this research, the levels of p-S6 Ser235/236 and p-Akt Ser473 were used to indicate the mTORC1 and mTORC2 activity, respectively (Ding et al., 2014). Prolonged rapamycin treatment could directly downregulate mTOR and Rictor (Sarbassov et al., 2006), which are both components of mTORC2 and essential to its functioning. To avoid the long-term effects of rapamycin in these experiments, we chose appropriate concentrations and durations to exert short-time effects. In our study, podocytes were treated with different concentrations of rapamycin for 24 h. Because there is no mTORC2-specific inhibitor available, a dual inhibitor of mTORC1 and mTORC2, AZD8055, was used to evaluate the effects of mTORC2 on BK channel expression (Willems et al., 2012). If BK channels were regulated by mTORC2, changes in BK channel levels would be detected after podocytes were exposed to AZD8055, but not rapamycin. Alternatively, if BK channels were only regulated by mTORC1, changes in BK channel levels would be detected after podocytes were exposed to AZD8055 and rapamycin. We found inhibiting mTORC1 using rapamycin and Raptor shRNA did not influence BK channels in podocytes. By contrast, inhibition of mTORC2 downregulated BK channel protein expression and current density in podocytes. This is the first demonstration that mTORC2, but not mTORC1, could mediate BK channel protein expression and activity. In Figure 4C, the currents density between control and rapamycin groups are differences in 100 and 110 mV voltage. But, there was no significant different decrease in NT shRNA and raptor shRNA groups. We speculate that these decreases may be caused by that the rapamycin could inhibit mTORC2 slightly.

Next, we studied the mechanisms by which mTORC2 regulates BK channels in podocytes. First, we suppressed Akt, the most important substrate of mTORC2 (Jhanwar-Uniyal et al., 2017). Interestingly, the Akt inhibitor MK2206 did not influence BK channel expression, but did cause a marked decrease in the current density of BK channels without influencing the voltage dependence or kinetics of the activation of podocyte BK channels. The results were consistent the previous research (Kim and Dryer, 2011). These data indicate mTORC2 mediated BK channel activity by influencing the surface expression of BK channels through Akt. Later, we suppressed PKCα and SGK1, two substrates of mTORC2, to test how mTORC2 mediated BK channel expression. We found inhibition of PKCα did not influence BK channel expression. However, inhibition of SGK1 caused a marked decrease in BK channel expression.

The precise role of BK channels in podocytes has not been established. However, BK channels directly interact with other SD proteins to influence the foot process and, as an ion channel, contribute to mechanosensation in foot processes and have gating properties that are sensitive to mechanical stretch of the plasma membrane (Kim et al., 2009a,b, 2010). Previous studies have shown insulin signaling acts through the PKGI (Piwkowska et al., 2015), Erk/MAPK, and PI3K/Akt signaling pathways to modulate mobilization of BK channels that is accompanied by a corresponding increase in current density (Kim and Dryer, 2011). This evidence indicates BK channels play a critical role in insulin-induced increases in glomerular barrier permeability and might participate in podocyte injury and proteinuria in diabetes.

Although the role of mTORC2 in renal diseases still needs clarification, increasing evidence has implicated mTORC2 in podocyte functions (Godel et al., 2011; Canaud et al., 2013; Eid et al., 2016; Zhang et al., 2016). Our study made a potential contribution to the molecular mechanism of the mTORC2 signaling pathway in regulation of the key ion channels, BK channels, in podocytes. In addition, a critical role for mTORC2 in podocyte function was also reported. Godel et al. (2011) found that mTORC2 was essential for podocyte adaptation and foot-process reorganization in diabetic nephropathy and Canaud et al. (2013) indicated that AKT2 activation by mTORC2 is required for podocyte viability and function in nephron reduction-induced renal disease.

Previous studies have shown that the BK inhibitor, Iberiotoxin, blocks insulin-induced disruption of the actin cytoskeleton (Piwkowska et al., 2015). Notably, mTORC2 is a key player in actin cytoskeleton involvement in podocyte integrity (Godel et al., 2011; Vassiliadis et al., 2011). In this regard, we speculate mTORC2 maybe regulate the actin cytoskeleton in podocytes via BK channels. BK channels are essential in regulating Ca^2+^ homeostasis, which mediates adaptive responses involved in podocyte function (Kim et al., 2009a). Recent data has shown protamine sulfate (PS) increases mTORC2 activity and Ca^2+^ influx, leading to damage of the glomerular filtration barrier and inhibition of Ca^2+^ influx, or mTORC2 is sufficient to block PS-induced damage to glomeruli (Vassiliadis et al., 2011). We speculate that part of the underlying reason for the increase in Ca^2+^ influx is PS upregulation of mTORC2 that increases BK channel activity and, thus, affords a large driving force for TRPC6 to efficiently transport Ca^2+^ into cells. This is important for further study of the role of BK channels and mTORC2 in kidney diseases.

In summary, our study demonstrated that inhibition of mTORC2 downregulates BK channel mRNA and protein levels and functions in podocytes. The complex mTORC2, a novel regulator of BK channels in podocytes, not only regulates the distribution of BK channels through Akt, but also modulates BK channel protein expression through SGK1, in podocytes.

## Author Contributions

YW and JT designed the research, performed the part of experiments, interpreted the data, and performed the data analysis. MW, FN, LY, XX, WZ, and HX performed the part of experiments. HC, KY, and XZ interpreted the data, drafted the manuscript, and revised it critically for intellectual content. All authors read and approved the final version of the manuscript before submission.

## Conflict of Interest Statement

The authors declare that the research was conducted in the absence of any commercial or financial relationships that could be construed as a potential conflict of interest.

## Footnotes

1 http://www.stanford.edu/%7Ecpatton/maxc.html
