# Supplementary material for: Mechanism of Regulation of Big-Conductance Ca2+-Activated K+ Channels by mTOR Complex 2 in Podocytes
Source: Front Physiol. 2019 Feb 28;10:167. doi: 10.3389/fphys.2019.00167 (PMC6403181; doi:10.3389/fphys.2019.00167)
Supplement: Supplementary file 1 [file Table_1.DOCX]

***Supplementary Material***

# Supplementary Materials and Methods

## Immunofluorescence

After cultured at 37℃ in medium lacking interferon-γ for 10-14 days, the differentiated podocytes were fixed in 4% paraformaldehyde in PBS for 15 minutes at room temperature. After rinsing twice with PBS, cells were blocked with 2% bovine serum albumen (BSA) in PBS for 1 hours at room temperature. Cells on coverglass were incubated with anti-synaptopodin antibody (1:500, ab224491, abcam), anti-podocin antibody (1:800, ab50339, abcam) and anti-nephrin anti body (1:500, ab216341, abcam) overnight at 4°C. Secondary antibodies were diluted in PBS with 2% BSA and cells were incubated at room temperature for 1 h. Alexa Fluor 555 goat anti-rabbit IgG (1:500; Cell Signaling Technology, USA) served as the secondary antibody. After rinsing three times with PBS containing 0.02% Tween 20, cells were incubated with DAPI (2 μg/ml) for 1 minute. Cells were rinsed three times with PBS containing 0.02% Tween 20 and cells on coverglass were mounted on microscope slides with Prolong Gold antifade reagent (Invitrogen, USA). Images were taken using a Carl Zeiss LSM710 confocal microscope and processed using Photoshop software (Adobe Systems, Inc., San Jose, CA).

# Supplementary Figures and Tables

## Supplementary Figure


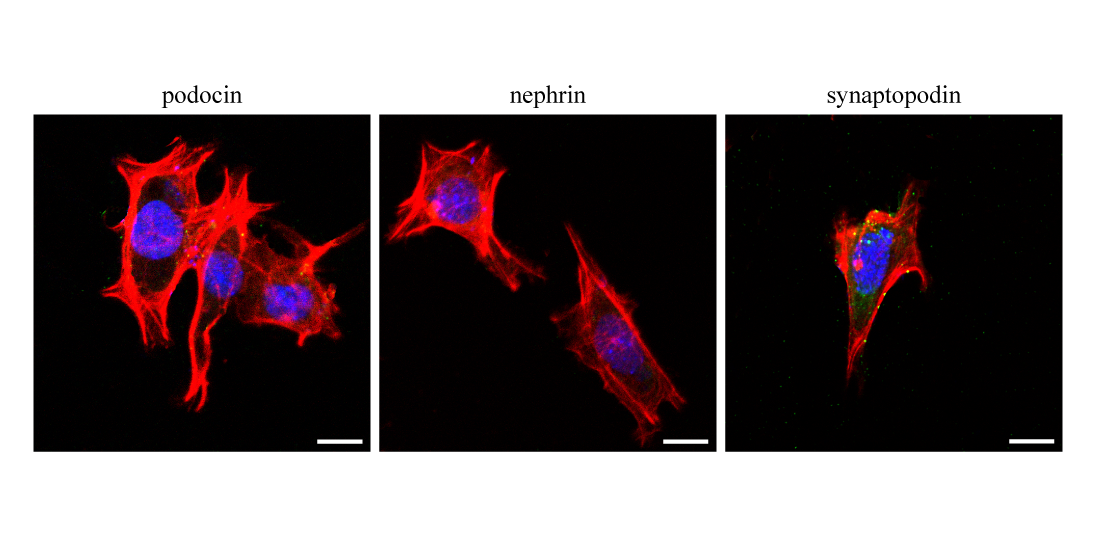


**Supplementary Figure 1.** Immunofluorescence microscopy of podocyte-specific marker proteins. After transferred to the temperature 37℃ 10-14 days, confocal microscopy of podocin, synaptopodin, and nephrin were expressed in differentiated podocytes, which showed these podocytes were able to maintain its original phenotype generated by Dr. Peter Mundel. Scale bars, 20 μm. Original magnification, ×630 (oil).
